# lncRNA PCGEM1 strengthens anti-inflammatory and lung protective effects of montelukast sodium in children with cough-variant asthma

**DOI:** 10.1590/1414-431X20209271

**Published:** 2020-06-05

**Authors:** Zhenxing Xu, Lingling Meng, Yuejuan Xie, Wei Guo

**Affiliations:** 1 Department of Pediatrics, The Affiliated Hospital of Yangzhou University, Yangzhou University, Jiangsu, Yangzhou, China; 2 Pulmonary Function Test Room of Children, The Affiliated Hospital of Yangzhou University, Yangzhou University, Jiangsu, Yangzhou, China

**Keywords:** Long non-coding RNA, PCGEM1, Montelukast sodium, Cough-variant asthma, Inflammation, NF-κB signaling pathway

## Abstract

Montelukast sodium is an effective and well-tolerated anti-asthmatic drug. Long non-coding RNAs (lncRNAs) are involved in the treatment of asthma. Therefore, this study aimed to investigate the effect of montelukast sodium on children with cough-variant asthma (CVA) and the role of lncRNA prostate cancer gene expression marker 1 (PCGEM1) in drug efficacy. The efficacy of montelukast sodium was evaluated by assessing the release of inflammatory factors and pulmonary function in CVA children after a 3-month treatment. An ovalbumin (OVA)-sensitized mouse model was developed to simulate asthmatic conditions. PCGEM1 expression in clinical peripheral blood samples and lung tissues of asthmatic mice was determined. Asthmatic mice experienced nasal inhalation of PCGEM1 overexpression with simultaneous montelukast sodium to investigate the roles of PCGEM1 in asthma treatment. The NF-κB axis after PCGEM1 overexpression was detected to explore the underling mechanisms. Consequently, montelukast sodium contributed to reduced levels of pro-inflammatory factors and improved pulmonary function in CVA children. PCGEM1 was poorly expressed in OVA-sensitized asthmatic mice and highly expressed in CVA children with response to the treatment. PCGEM1 overexpression enhanced the anti-inflammatory effects and promoted effects on pulmonary function of montelukast sodium in CVA children and OVA-sensitized asthmatic mice. Furthermore, PCGEM1 inhibited the activation of the NF-κB axis. This study demonstrated the anti-inflammatory and lung-protective effects of montelukast sodium on CVA, which was strengthened by overexpression of PCGEM1. Findings in this study highlighted a potential anti-asthmatic target of montelukast sodium.

## Introduction

Asthma is a disorder with symptoms including wheeze, dyspnea, and cough. However, the presentation of cough-variant asthma (CVA) is atypical, with cough being the only or main symptom (1). CVA is one of the inducers of chronic cough in China (2). Additionally, mucus secretory response is also present in patients with CVA (3). Airway inflammation as well as remodeling characterized with subbasement membrane thickening exhibits associations with CVA and non-asthmatic chronic cough (4). Also, eosinophils and neutrophils are possibly activated and implicated in the pathophysiology of CVA (5). Children with CVA have a high prevalence of inaudible wheezing that may be diminished by inhalation of β2 agonist (6). Montelukast sodium, a potent leukotriene 1 receptor antagonist, has been shown to control chronic non-productive cough effectively in CVA (7). Montelukast sodium has also been applied alone or combined with other anti-asthmatic drugs like budesonide, which is superior to budesonide alone for treating the children with chronic CVA (8). However, little attention has been paid to the mechanisms underlying the therapeutic effect of montelukast sodium on asthma and CVA. From a clinical perspective, long noncoding RNAs (lncRNAs) are possibly of great value as biomarkers for classification and/or efficacy assessment of drugs in respiratory diseases (9).

lncRNAs are a group of RNAs with more than 200 nucleotides that can regulate physiological functions and influence disease development in various organ systems, such as cardiovascular, endocrine, digestive, respiratory, etc. (10). Several of them are dysregulated after asthma induction and some are co-expressed with inflammatory cytokines and receptors that are involved in airway allergic inflammation (11). For instance, lncRNA plasmacytoma variant translocation 1 (PVT1) regulates the proliferation of airway smooth muscle cells and release of IL-6 in the patients suffering from severe asthma (12). A prostate-specific lncRNA prostate cancer gene expression marker 1 (PCGEM1) is a powerful indicator that distinguishes early osteoarthritis from late-stage osteoarthritis (13). lncRNA PCGEM1 is reported to be a mediator of multiple metabolic pathways, such as glucose and glutamine metabolism (14). A preliminary screening of dysregulated lncRNAs presents a low expression of PCGEM1 in the serum samples of patients with asthma (15). In this study, we aimed to investigate the significance of PCGEM1 in the treatment with montelukast sodium for children with CVA, so as to develop novel therapies that can be better targeted toward CVA-specific characteristics.

## Material and Methods

### Ethics statement

Experiments involving human beings were conducted with approval of the Ethics Committee of the Affiliated Hospital of Yangzhou University and in line with the Declaration of Helsinki. Informed consents were obtained from the relatives of all participants. Animal experiments were performed in compliance with the recommendations of the Guide for the Care and Use of Laboratory Animals of the National Institutes of Health. The protocol was approved by the Animal Ethics Committee of the Affiliated Hospital of Yangzhou University.

### Study subjects

A total of 60 children diagnosed with CVA from March 2017 to March 2018 were enrolled in this study, including 33 males and 27 females, aged from 2 to 9 years (mean age 5.5±1.7 years). Disease severity was defined by the Global Initiative for Asthma (GINA) 2016 (http://www.ginasthma.com), and all children were defined with mild asthma (GINA 1 or 2). The disease duration ranged from 7 months to 21 months, with a mean duration of 14.4±2.3 months. Additionally, 60 healthy children were enrolled as normal controls, with 31 males and 29 females, aged from 1 to 10 years with a mean age of 5.9±1.4 years. Children with other types of respiratory tract infections and hormonal contraindications were excluded. All the patients received standard treatment for the management of an acute attack of bronchial asthma as per the GINA guidelines. These included parenteral steroids, short-acting beta 2 agonists with inhaled anti-cholinergics by nebulization every 4–6 hourly depending on severity, intravenous theophylline derivatives, oxygen therapy, and other supportive therapy (16). They were administered with one piece of montelukast sodium (BaiSanPing; 10 mg, orally) (China OTSUKA Pharmaceutical Co. Ltd., China; SFDA approval number: H20064370) daily at bedtime. One month indicated one course, and children experienced 3 consecutive courses (3 months).

### Measurement of inflammatory cytokines and evaluation of pulmonary function

Pulmonary function was evaluated before and after treatment and three months after treatment, the levels of inflammatory factors in the peripheral blood of children were measured. Fasting venous blood was collected before and after treatment, and interleukin (IL)-4, IL-3, and interferon (IFN)-γ levels were measured by enzyme-linked immunosorbent assay (ELISA) kits (Shanghai ExCell Biological Product Co., Ltd., China). Pulmonary function indicators including peak expiratory flow (PEF), forced vital capacity (FVC), instantaneous maximum expiratory flow after 50% expiration of the FVC (MEP50), and forced expiratory volume in first second (FEV_1_) were recorded by a pulmonary function detector (MIR Spirolab III srl, Italy) before and after treatment.

### Evaluation of therapeutic effects

Clinical efficacy was evaluated based on the Chinese guidelines for the diagnosis and prevention of childhood bronchial asthma (17) 3 months after treatment. Remarkably effective cases: basically no episode of shortness of breath and wheezing, occasional or no cough; valid cases: asthma attack but significantly reduced frequency (<2 times/week); invalid cases: no significant amelioration in the frequency or severity of asthma attacks. In the subsequent experiments, the effective and remarkably effective cases were classified as valid, and the rest were classified as invalid. The expression of lncRNA PCGEM1 in these two groups was determined.

### Mouse model of CVA

Thirty male Balb/c mice aged 6–8 weeks were purchased from the Experimental Animal Center of Xi'an Jiaotong University Health Science Center and were acclimated for 3 days before the experiment. Twenty-four mice were randomly selected for asthma model construction and the remaining 6 mice were selected for normal control (MOCK).

An asthma mouse model was established by ovalbumin (OVA) sensitization (Sigma Chemical, USA). In details, each mouse was intraperitoneally injected with 200 μL phosphate buffer saline (PBS) containing 50 μg OVA and 2 mg aluminum hydroxide gel on the 1st and 14th day, followed by atomization inhalation with OVA for 20 minutes from the 21st to 26th day to establish an asthma model (18).

Among the 24 mice with OVA-induced asthma, 6 mice were randomly selected and intraperitoneally administered montelukast sodium (3 mg/kg; (19)) 30 min before atomization inhalation from the 21st to 26th day, for a total of 7 times. Another 6 OVA-sensitized mice experienced nasal inhalation of PCGEM1 negative control (NC) plasmid (4 mg/kg) and montelukast sodium treatment from the 21st to 26th day, for a total of 7 times (20,21). Another 6 OVA-sensitized mice experienced nasal inhalation of PCGEM1 mimic plasmid (4 mg/kg; Shanghai GenePharma Co., Ltd, China) and montelukast sodium treatment from the 21st to 26th day.

### Assessment of bronchial hyperresponsiveness

Total airway resistance in mice was assessed 24 h after the last atomization inhalation by a mouse pulmonary function instrument (RC System, Buxco Electronics Inc., USA). The spontaneous respiration of the mice in the awake state was recorded by plethysmography. The mice were atomized with methacholine at different concentrations (0, 6.25, 12.5, 25, and 100 mg/mL) for 3 min, and connected with a MP150 multichannel physiological recorder (BIOPAC Systems, Inc., Goleta, USA). The data were recorded and averaged. The data are reported as increased airway resistance (Penh).

### Pulmonary function test

The mice were anesthetized with 5% pentobarbital sodium solution, after which their limbs were fixed and cervical subcutaneous tissues were separated. The trachea was exposed and intubated to a ventilator (HX-300, Chengdu Techman Software Co., Ltd., China) with a respiratory frequency of 90 times/min, a tidal volume of 5.6 mL/kg, and respiratory pressure of −8 cmH_2_O (1 cmH_2_O=0.098 kPa). The dynamic transpulmonary pressure, flow, and tidal volume were recorded. PEF and the ratio of forced expiratory volume in 0.4 s (FEV0.4) to FVC were calculated when the ventilation was performed with 5 times the tidal volume, and expiration was assisted with −8 cmH_2_O pressure.

### Cell counting in bronchoalveolar lavage fluid (BALF)

The mice were intubated and fixed after anesthesia. BALF was performed with ice-cold PBS, 0.8 mL/time for 3 times. BALF was collected and centrifuged at 500 *g* at 4°C for 5 min. The supernatant was collected and stored at low temperature for later use. The total inflammatory cells, lymphocytes, macrophages, neutrophils, and eosinophils were counted by Wright's staining (Beijing Solarbio Technology Co., Ltd., China) after lysis of red blood cells.

### Hematoxylin-eosin (HE) staining

The blood of the lung surface was washed with ice-cold PBS buffer after sterilization. The left lung was fixed in 10% neutral formalin for 24 h, routinely embedded in paraffin and sectioned at 4 μm for HE staining, periodic acid-schiff (PAS) staining (Beijing Solarbio Technology Co., Ltd.), and immunofluorescence to observe the pathological changes of lung tissues in mice. The right lung was preserved in an ultra-low temperature refrigerator.

### RT-qPCR

Total RNA was extracted from the lung tissues using TRIzol kit (Invitrogen, USA). The concentration and purity of total RNA were determined using a Nanodrop 2000 ultramicro spectrophotometer (Thermofisher Scientific, UK). Then cDNA was synthesized using the reverse transcription kit (GeneCopeia, USA). The expression of each gene in Table 1 was detected using SYBR PCR Master Mix kit (Applied Biosystems, USA) on the PCR system (Applied Biosystems). With β-actin as the internal reference, the relative expression of the gene was expressed by 2^-ΔΔCt^. All primers were synthesized by Shanghai Biotechnology (Shanghai, China).

### ELISA

The contents of inflammatory cytokines IL-4, IL-13, and IFN-γ were measured according to the instructions of ELISA kits (Shanghai ExCell Biological Product Co., Ltd., China).

### Western blot assay

The lung tissues frozen at ultra-low temperature were thawed on ice and lysed by radio-immunoprecipitation assay (RIPA) lysis buffer. After homogenization, the supernatant was collected by centrifugation (14,000 *g*, 15 min, 4°C) to obtain total protein. After the concentration of the protein was measured with the bicinchoninic acid (BCA) protein assay kit (TransGen Biotech, China), 40 μg of total protein per well was subjected to sodium dodecyl sulfate-polyacrylamide gel electrophoresis (SDS-PAGE). The separated proteins were transferred onto the nitrocellulose membrane. After being blocked with 5% skim milk powder, the membrane was incubated with antibody to NF-κB (1:1000, ab220803, Abcam, USA) and p-NF-κB (1:1000, ab194908, Abcam) at 4°C overnight, followed by TBST (tris-buffered saline and Tween 20) washing. Next, the membrane was incubated with secondary antibody at room temperature for 45 min, followed by TBST washing. The membrane was stained with electrochemiluminescence (ECL) liquid and photographed. Grayscale analysis was performed to analyze the absorbance value.

### Immunofluorescence assay

After dewaxing and dehydration with gradient alcohol, antigen repair of lung tissues was carried out. The tissue sections were washed with 0.01M PBS 3 times (5 min/time) and blocked with 2% bovine serum albumin at room temperature for 20 min, and incubated with the primary antibody NF-κB (ab7204) at 4°C overnight. After thorough PBS washing, the sections were incubated with secondary antibody horseradish peroxidase (ab205718) for 40 min. The sections were washed thoroughly with PBS and sealed with glycerin. The fluorescence was observed under fluorescence microscope (BX51; Olympus, Japan).

### Statistical analysis

Data were analyzed by SPSS 21.0 statistical software (IBM, USA). The data normality was tested by Kolmogorov-Smirnov test. The data are reported as means±SD. Comparisons between two groups were analyzed by *t*-test, and among multi-groups by one-way analysis of variance (ANOVA) or two-way ANOVA. The *post hoc* test was performed by Sidak's multiple comparisons test or Tukey's multiple comparisons test. The receiver-operating characteristics (ROC) curve was drawn to evaluate the diagnostic value of PCGEM1 expression for the efficacy of montelukast sodium. A two-tailed P value less than 0.05 indicated statistically significant difference.

## Results

### Montelukast sodium reduced inflammation and improved pulmonary function in CVA children

**Figure 1:**
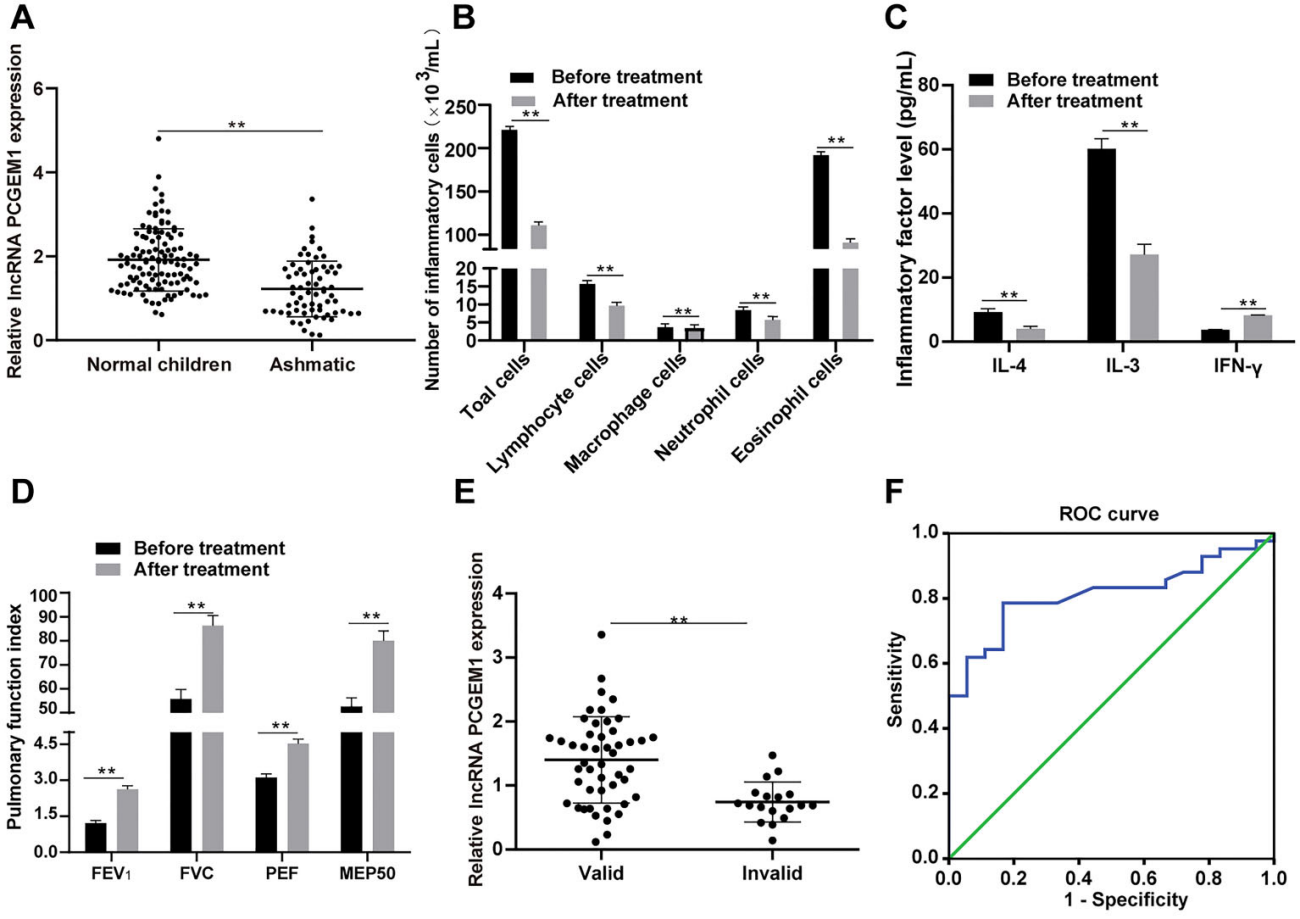
Montelukast sodium exerts inhibitory effects on inflammation and promotive effect on pulmonary function in cough-variant asthma (CVA) children. **A**, RT-qPCR determination of lncRNA prostate cancer gene expression marker 1 (PCGEM1) expression in peripheral blood lymphocytes of CVA children (n=60) and normal children. **B**, The number of total peripheral blood inflammatory cells, lymphocytes, macrophages, neutrophils, and eosinophils in CVA children. **C**, The levels of inflammatory mediators in peripheral blood of CVA children measured by ELISA. **D,** Evaluation of pulmonary function index of CVA children: forced expiratory volume in first second (FEV_1_), forced vital capacity (FVC), peak expiratory flow (PEF), and maximum expiratory flow after 50% expiration of the FVC (MEP50). **E**, RT-qPCR determination of lncRNA PCGEM1 expression in children with different efficacy 3 months after treatment. **F**, ROC curve analysis of the diagnostic value of PCGEM1 for asthma; sensitivity=78.6%, specificity=77.8%. Data are reported as means±SD. All experiments were repeated 3 times. **P<0.05, data in panels **A** and **E** were analyzed by the independent *t*-test, in panels **B**, **C,** and **D** by two-way ANOVA, followed by Sidak's multiple comparisons post hoc test, and in panel **F** by ROC curve.

lncRNAs are reported to be involved in the regulation of inflammatory mediators or the expression of cytokines (22). lncRNA PCGEM1 is lowly expressed in the serum of asthma patients (15). Therefore, we speculated that PCGEM1 may affect the treatment of asthma patients. lncRNA PCGEM1 expression was markedly reduced in asthmatic children compared to normal children (P<0.05; Figure 1A). In addition, the numbers of inflammatory cells, lymphocytes, macrophages, neutrophils, and eosinophils in the peripheral blood of children with CVA were all significantly reduced (P<0.05). As shown by ELISA, IL-4 and IL-3 levels were remarkably decreased, and IFN-γ level was elevated after montelukast sodium treatment (P<0.05). The levels of PEF, FVC, FEV_1_ and MEP50 were increased by montelukast sodium treatment (P<0.05). After 3 months of treatment, CVA children were assigned to response group or non-response group, and PCGEM1 expression was markedly increased in the response cases (P<0.05). Further, the ROC curve analysis showed that PCGEM1 had a diagnostic value for asthma. The area under the curve was 0.813, with a sensitivity of 78.6% and a specificity of 77.8% (Figure 1B–F).

**Figure 2:**
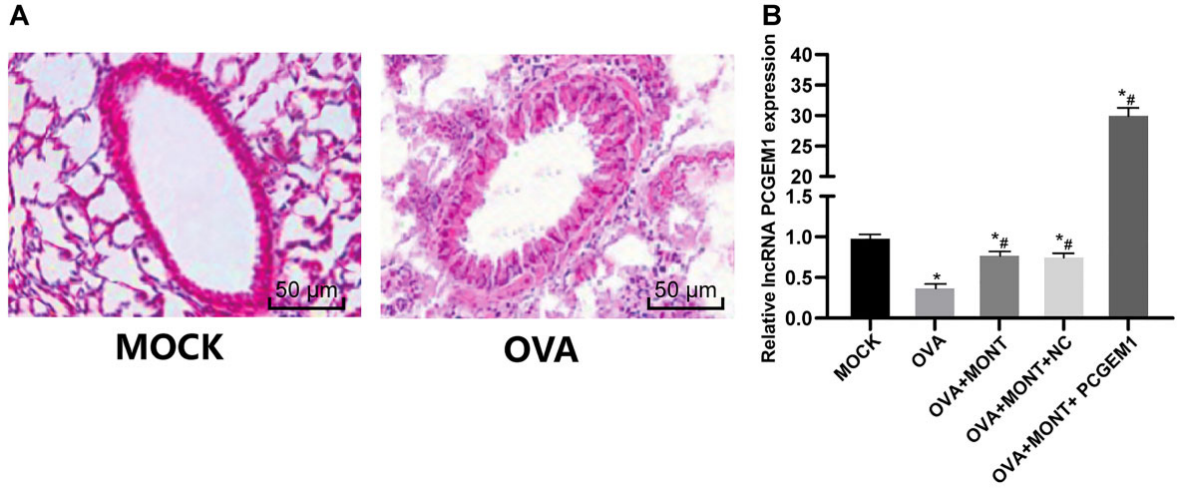
Prostate cancer gene expression marker 1 (PCGEM1) was expressed at a low level in ovalbumin (OVA)-sensitized asthmatic mice and normal control (MOCK). **A**, Infiltration of inflammatory cells in the lung tissues of asthmatic and control mice detected by HE staining (n=3, scale bar: 50 μm); **B**. RT-qPCR determination of lncRNA PCGEM1 expression in lung tissues of untreated asthmatic mice (n=3) and after treatment with montelukast sodium (MONT) and PCGEM1. Data are reported as means±SD. *P<0.05 *vs* the MOCK group; ^#^P<0.05 *vs* the OVA group (one-way ANOVA, followed by Tukey's multiple comparisons *post hoc* test). NC: negative control.

**Figure 3:**
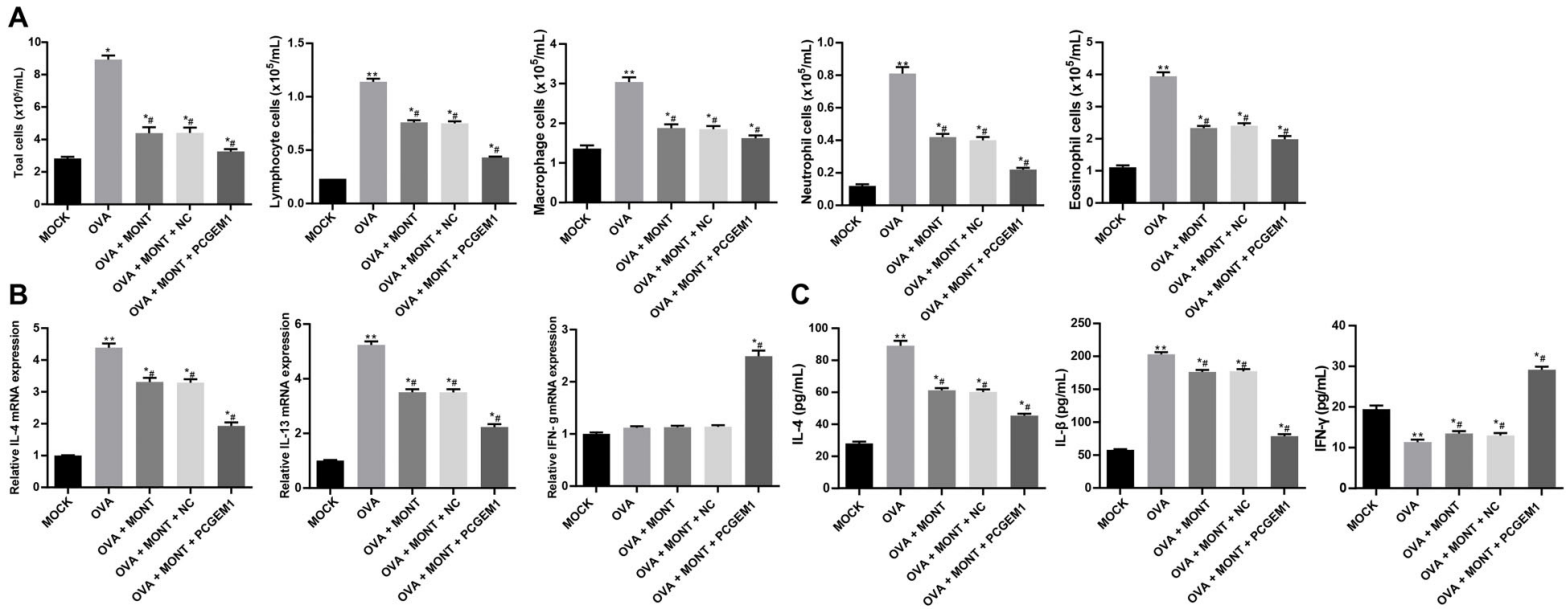
Upregulation of prostate cancer gene expression marker 1 (PCGEM1) strengthened the inhibitory effects of montelukast sodium on inflammation in ovalbumin (OVA)-sensitized asthmatic mice. **A**, The number of total inflammatory cells in the bronchoalveolar lavage fluid of asthmatic mice (n=6) treated with montelukast sodium (MONT) and PCGEM1, and normal control (MOCK). **B**, mRNA expression of inflammatory mediators in lung tissues of animals treated with montelukast sodium and PCGEM1 mimic determined by RT-qPCR (n=3). **C**, The levels of inflammatory mediators in the lung tissues of animals measured by ELISA (n=6). Data are reported as means±SD. *P<0.05, **P<0.01 *vs* the MOCK group; ^#^P<0.05 *vs* the OVA group (one-way ANOVA, followed by Tukey's multiple comparisons *post hoc* test). NC: negative control.

**Figure 4:**
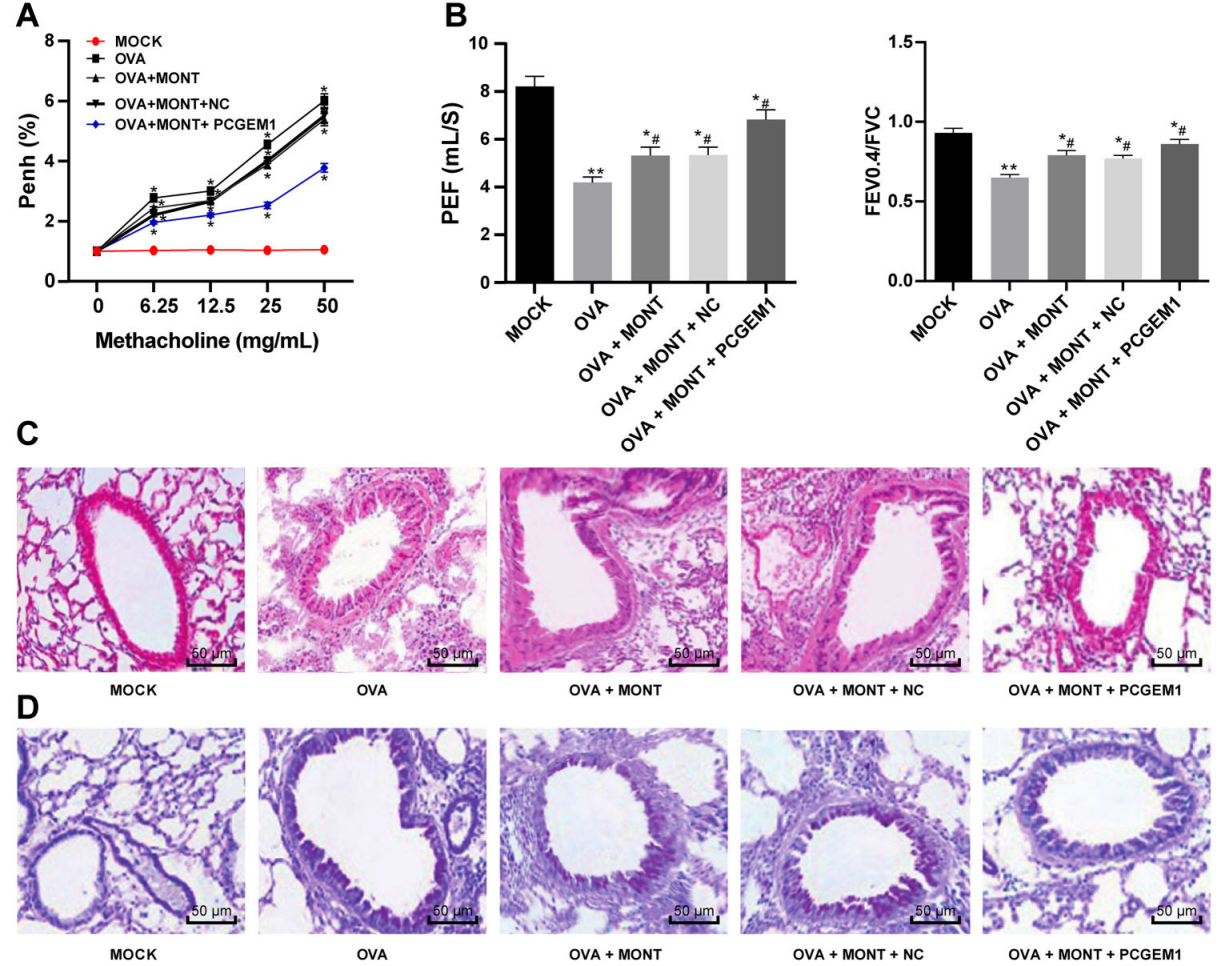
Upregulation of prostate cancer gene expression marker 1 (PCGEM1) strengthened the effects of montelukast sodium on pulmonary function in ovalbumin (OVA)-sensitized asthmatic mice treated with montelukast sodium (MONT) and PCGEM1 mimic, and normal control (MOCK). **A**, The airway hyperresponsiveness in the animals (n=6). **B**, Evaluation of peak expiratory flow (PEF) and forced expiratory volume in 0.4 s (FEV0.4) to forced vital capacity (FVC) ratio in the animals (n=6). **C**, HE staining showing inflammatory cell infiltration in lung tissue of the animals (n=3). **D**, PAS staining showing mucin secretion in airway goblet cells of the animals (n=3). Data are reported as means±SD. *P<0.05, **P<0.001 *vs* the MOCK group; ^#^P<0.05 *vs* the OVA group. Data in panel **A** were analyzed by one-way ANOVA, followed by Tukey's multiple comparisons *post hoc* test. Data in panel **B** were analyzed by two-way ANOVA, followed by Tukey's multiple comparisons *post hoc* test. PENH(%): increased airway resistance.

**Figure 5:**
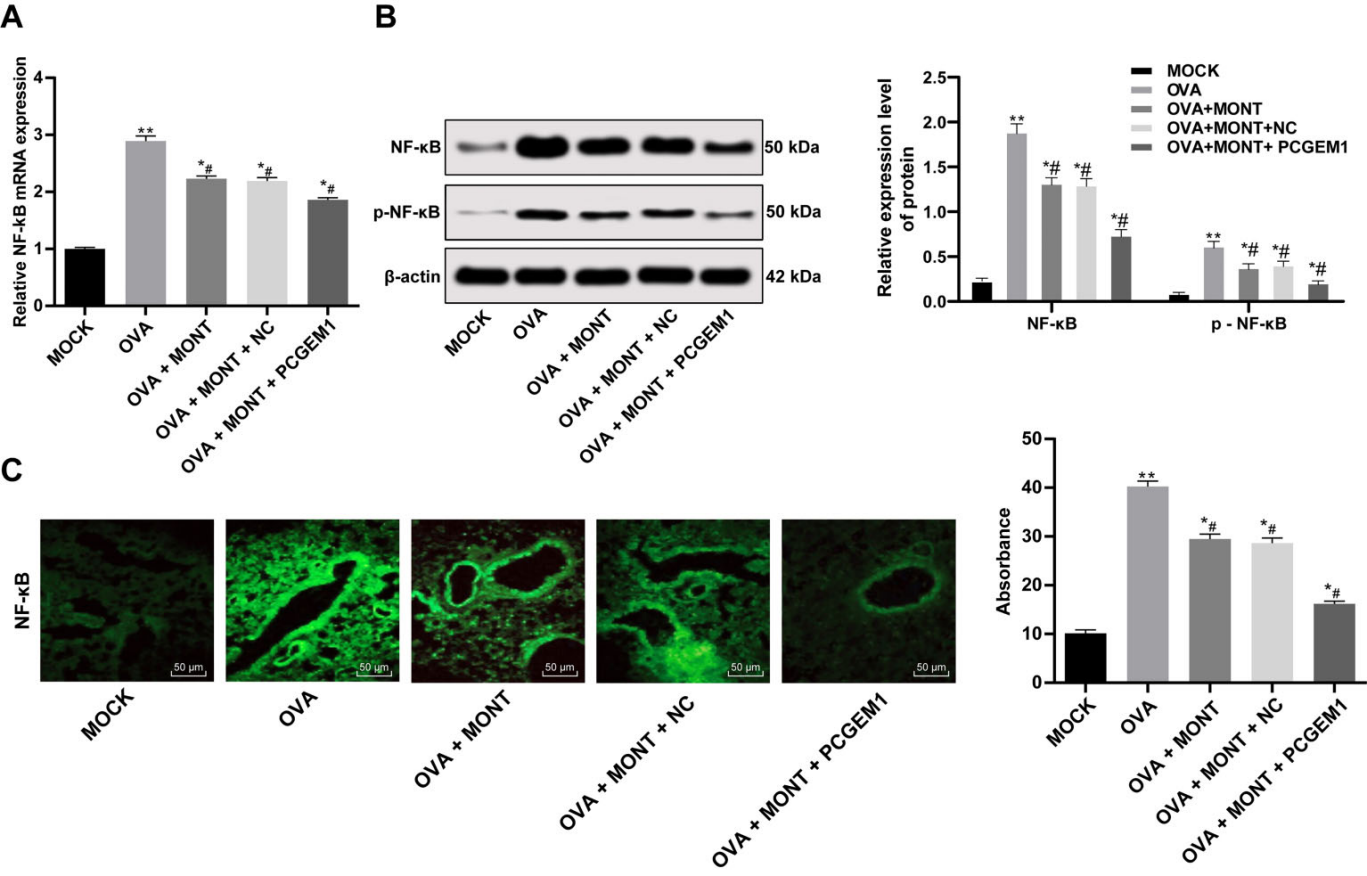
Upregulation of prostate cancer gene expression marker 1 (PCGEM1) combined with montelukast sodium reduced the NF-κB signaling in the ovalbumin (OVA)-sensitized asthmatic mice treated with montelukast sodium (MONT) and PCGEM1 mimic, and normal control (MOCK). **A**, RT-qPCR determination of NF-κB mRNA expression in the lung tissues. **B**, Western blot assay of NF-κB and p-NF-κB expression in the lung tissues. **C**, Immunofluorescence analysis of NF-κB expression in the lung tissue. Data are reported as means±SD. *P<0.05, **P<0.001 *vs* the MOCK group; ^#^P<0.05 *vs* the OVA group. Data in panel **A** were analyzed by one-way ANOVA, and data in panels **B** and **C** were analyzed by two-way ANOVA, followed by Tukey's multiple comparisons *post hoc* test. NC: negative control.

**Table 1:** Primer sequences for RT-qPCR.

Gene	Primer sequence
lncRNA PCGEM1	F: ACCTTTTTGCCCTATGCCGT
	R: ACGTTGAGTCCCAGTGCATC
IL-4	F: GTCACTCTGCTCTTCTTTCTCG
	R: CTCTCTGTGGTGTTCTTCGTTG
IL-13	F: TCTTGCTTGCCTTGGTGGTCTC
	R: TCCTCATTAGAAGGGGCCGTG
IFN-γ	F: TCAAGTGGCATAGATGTGGAA
	R: TGGCTCTGCAGGATTTTCATG
NF-κB	F: GGCCGGAAGACCTATCCTACT
	R: CTACAGACACAGCGCACACT
β-actin	F: GTCATTCCAAATATGAGAGATGCGT
	R: GCTATCACCTCCCCTGTGTG

lncRNA: long non-coding RNA; PCGEM1: prostate cancer gene expression marker 1; IL-4: interleukin-4; IL-13: interleukin-13; IFN-γ: interferon γ; NF-κB: nuclear factor κB; F: forward; R: reverse.

### PCGEM1 was lowly expressed in OVA-sensitized asthmatic mice

The OVA-sensitized asthmatic mice manifested frequent itching, sneezing, and accelerated breathing. The lung tissue of the mouse presented inflammatory cell infiltration, destroyed alveolar structures, and mild bleeding. Meanwhile, the alveolar cavity, bronchus, and peripheral blood vessels were filled with lymphocytes and eosinophils (Figure 2A). The expression of PCGEM1 in lung tissues of asthmatic mice was decreased (P<0.05), which was significantly increased after nasal administration of PCGEM1 mimic (P<0.05; Figure 2B).

### PCGEM1 overexpression enhanced the inhibitory effects of montelukast sodium on inflammation in OVA-sensitized asthmatic mice

More inflammatory cells, lymphocytes, macrophages, neutrophils, and eosinophils were observed in the BALF of asthmatic mice than the MOCK mice (P<0.05); whereas, those were reduced in the BALF of asthmatic mice treated with montelukast sodium and further reduced in the BALF of asthmatic mice treated with montelukast sodium and administered PCGEM1 mimic (P<0.05; Figure 3A).

The levels of IL-4 and IL-13 in the lung tissues of asthmatic mice were significantly increased (both P<0.05), and the levels of IFN-γ were not noticeably changed compared to the MOCK mice. The levels of IL-4 and IL-13 were decreased in the lung tissues of asthmatic mice treated with montelukast sodium and administered PCGEM1 mimic (P<0.05; Figure 3B and C). To conclude, PCGEM1 overexpression contributed to suppression of inflammation in OVA-sensitized asthmatic mice following montelukast sodium treatment.

### PCGEM1 overexpression enhanced the promotive effects of montelukast sodium on pulmonary function in OVA-sensitized asthmatic mice

With the increase of the concentration of methacholine, the airway hyperresponsiveness of asthmatic mice increased and the lung function index FEV_0.4_/FVC decreased (P<0.05), but reduced airway hyperresponsiveness and increased FEV_0.4_/FVC level were detected in the asthmatic mice treated with montelukast sodium (P<0.05). More noticeable reduction in airway hyperresponsiveness and increase in FEV_0.4_/FVC level were found in the asthmatic mice treated with montelukast sodium and PCGEM1 mimic (Figure 4A and B).

HE and PAS staining assays showed disordered lung tissue, thickened alveolar wall, increased inflammatory cell infiltration around the bronchia, increased mucus secretion, and goblet cell hyperplasia in the asthmatic mice. After treatment with montelukast sodium, the lung tissue structure of the asthmatic mice was slightly improved, and mucin secretion and the number of inflammatory cells around goblet cells and bronchi were reduced. After montelukast sodium combined with PCGEM1 overexpression, the lung tissue structure of asthmatic mice was significantly improved, and the number of inflammatory cells around the bronchi was significantly reduced (Figure 4C and D). As a result, PCGEM1 overexpression contributed to improvement in the pulmonary function in OVA-sensitized asthmatic mice following montelukast sodium treatment.

### PCGEM1 overexpression plus montelukast sodium blocked the NF-κB signaling pathway in the OVA-sensitized asthmatic mice

Nuclear factor-κB (NF-κB) signaling plays an important role in the inflammatory response, and is activated in chronic obstructive pulmonary disease tissues (23). We speculated that PCGEM1 may mediate the expression of NF-κB. It was found that NF-κB mRNA expression, NF-κB protein expression, p-NF-κB expression, and NF-κB fluorescence (Figure 5A–C) were increased in the lung tissues of asthmatic mice, but was reduced in lung tissues of asthmatic mice treated with montelukast sodium and PCGEM1 mimic (P<0.05). This suggested that lncRNA PCGEM1 may enhance the therapeutic effect of montelukast sodium on asthma by blocking the NF-κB signaling pathway.

## Discussion

Airway inflammation attributed to eosinophil and T-lymphocyte infiltration is involved in asthma (24). The exploration of underlying mechanisms associated with asthma phenotypes could possibly lead to better molecule-targeted therapies and improved disease prognosis (25). Our study principally evaluated the role of PCGEM1 in the treatment of montelukast sodium for CVA and a ROC showed a good diagnostic value of PCGEM1. Animal experiments were conducted to further investigate the effects of PCGEM1 combined with montelukast sodium on the inflammation and lung function of in an OVA-induced asthmatic murine model. The data showed that PCGEM1 strengthened the anti-inflammatory and lung protective effects of montelukast sodium in OVA-sensitized asthmatic mice.

Our data showed that montelukast sodium induced an anti-inflammatory response by increasing the level of IFN-γ together with reducing the levels of IL-3 and IL-4 and the numbers of total inflammatory cells, lymphocytes, macrophages, neutrophils, and eosinophils in the peripheral blood of children with CVA. Consistently, montelukast sodium treatment contributes to a reduction in the levels of IL-4 and IL-13, and airway inflammatory cell infiltration in an OVA-challenged murine model (26). Meanwhile, montelukast sodium suppresses eosinophilic inflammation in airways in mild asthmatic children (27). Montelukast sodium statistically elevates the serum level of anti-inflammatory protein IL-10 and reduces peripheral blood eosinophils following a 4-week treatment, providing clinical benefits for children with chronic asthma (28). As demonstrated by RT-qPCR determination, PCGEM1 was lowly expressed in CVA patients and its expression was elevated after montelukast sodium treatment and in the valid cases of CVA. lncRNAs are able to regulate inflammation by mediating recognized inflammatory mediators including TNF-α, IL-1, IL-6, and IL-18, as well as cell adhesion molecules such as ICAM-1 and VCAM-1 (29). IL-3-producing basophils that could release IL-4, IL-6, and IL-13 triggered exacerbation of airway hyperresponsiveness in a murine inflammatory model (30). IFN-γ causes reductions in type 2 innate lymphoid cell (ILC2) expansion and IL-13 expression thereby blocking the progression of asthma-like phenotype (31). This study confirmed the enhanced anti-inflammatory effects of montelukast sodium mediated by upregulation of PCGEM1, evidenced by inhibited inflammation in the BALF and reduced IL-4 and IL-13 levels and increased IFN-γ level in the lung tissues.

In addition, a 3-month montelukast sodium treatment improved pulmonary function by elevating pulmonary function indices PEF, FVC, FEV_1_, and MEP50. In the murine model of asthma, montelukast sodium reduced airway hyperresponsiveness and increased FEV_0.4_/FVC identically. Pulmonary function indices, FEV_1_, and PEF are the main markers of the severity of asthma and response to the treatment. Montelukast sodium elevated FEV_1_ after 2 h (32), and simple montelukast sodium for children with CVA contributed to elevations in PEF and FEV_1_ after treatment and at follow-up (33), with which our results are in line. Airway hyperresponsiveness and inflammation are causes of airway remodeling in asthma, which could be reversed by montelukast sodium (34). The *in vivo* experiments demonstrated that upregulation of PCGEM1 strengthened the protective effects of montelukast sodium on pulmonary function by reducing airway hyperresponsiveness and increasing FEV_0.4_/FVC.

Finally, upregulation of PCGEM1 combined with montelukast sodium treatment blocked the activation of NF-κB mRNA and protein after asthma induction. Inhibited NF-κB activation that mediates the production of IL-6, TNF-α, and MCP-1 is also required for the anti-inflammatory effect of montelukast sodium (35). Chrysophanol, another anti-asthmatic drug, ameliorates airway inflammation and remodeling by inhibiting the NF-κB signaling pathway (36). Montelukast sodium reduces Aβ1-42-induced toxicity to primary neurons via targeting CysLT1R-mediated NF-κB signaling (37). lncRNAs interplay with mRNA and each other to control the mRNA stability and/or translation involved with lung inflammation (38). In a similar way, lncRNA HOTAIR alleviates inflammation in rheumatoid arthritis by targeting the NF-κB signaling pathway (39). Based on the above findings, it is reasonable to conclude that lncRNA PCGEM1 potentially enhances the therapeutic effect of montelukast sodium on asthma by blocking the NF-κB signaling pathway.

Taken together, our findings demonstrated that overexpression of PCGEM1 potentiated the anti-inflammatory effect of montelukast sodium and promotive effect on pulmonary function, which highlights a novel therapeutic approach for children with CVA. Also, we suggest that lncRNA PCGEM1 potentiated the therapeutic effect of montelukast sodium on asthma by blocking the NF-κB signaling pathway.

However, the blockade of NF-κB activation in this process remains to be confirmed with further pathway inhibitor experiments. No negative control group of montelukast sodium was included in our experimental study, which may affect our results to some degree. In the near future, further research will be carried out to discuss the molecular mechanism in CVA.

## References

[1] Morjaria JB, Kastelik JA (2011). Unusual asthma syndromes and their management. Ther Adv Chronic Dis.

[2] Lai K, Chen R, Lin J, Huang K, Shen H, Kong L (2013). A prospective, multicenter survey on causes of chronic cough in
China. Chest.

[3] Rubin BK, Priftis KN, Schmidt HJ, Henke MO (2014). Secretory hyperresponsiveness and pulmonary mucus
hypersecretion. Chest.

[4] Matsumoto H, Niimi A, Tabuena RP, Takemura M, Ueda T, Yamaguchi M (2007). Airway wall thickening in patients with cough variant asthma and
nonasthmatic chronic cough. Chest.

[5] Matsuoka H, Niimi A, Matsumoto H, Takemura M, Ueda T, Yamaguchi M (2010). Inflammatory subtypes in cough-variant asthma: association with
maintenance doses of inhaled corticosteroids. Chest.

[6] Enseki M, Nukaga M, Tadaki H, Tabata H, Hirai K, Kato M (2019). A breath sound analysis in children with cough variant
asthma. Allergol Int.

[7] Kita T, Fujimura M, Ogawa H, Nakatsumi Y, Nomura S, Ishiura Y (2010). Antitussive effects of the leukotriene receptor antagonist
montelukast in patients with cough variant asthma and atopic
cough. Allergol Int.

[8] Wang XP, Yang LD, Zhou JF (2018). Montelukast and budesonide combination for children with chronic
cough-variant asthma. Medicine (Baltimore).

[9] Booton R, Lindsay MA (2014). Emerging role of MicroRNAs and long noncoding RNAs in respiratory
disease. Chest.

[10] Kong Y, Lu Z, Liu P, Liu Y, Wang F, Liang EY (2019). Long noncoding RNA: genomics and relevance to
physiology. Compr Physiol.

[11] Wang SY, Fan XL, Yu QN, Deng MX, Sun YQ, Gao WX (2017). The lncRNAs involved in mouse airway allergic inflammation
following induced pluripotent stem cell-mesenchymal stem cell
treatment. Stem Cell Res Ther.

[12] Austin PJ, Tsitsiou E, Boardman C, Jones SW, Lindsay MA, Adcock IM (2017). Transcriptional profiling identifies the long noncoding RNA
plasmacytoma variant translocation (PVT1) as a novel regulator of the
asthmatic phenotype in human airway smooth muscle. J Allergy Clin Immunol.

[13] Zhao Y, Xu J (2018). Synovial fluid-derived exosomal lncRNA PCGEM1 as biomarker for
the different stages of osteoarthritis. Int Orthop.

[14] Hung CL, Wang LY, Yu YL, Chen HW, Srivastava S, Petrovics G (2014). A long noncoding RNA connects c-Myc to tumor
metabolism. Proc Natl Acad Sci USA.

[15] Li BW, Gao Z, Liang HY, Jiang XF (2016). Preliminary screening of differential expression of circulating
lncRNA in serum of asthmatic patients [in Chinese]. J Med Res.

[16] Chaudhury A, Gaude GS, Hattiholi J (2017). Effects of oral montelukast on airway function in acute asthma: a
randomized trial. Lung India.

[17] Respiratory Group of Pediatric Society of Chinese Medical
Association, Editorial Board of Chinese Journal of Pediatrics (2016). Guidelines for the diagnosis and prevention of childhood
bronchial asthma (2016 Edition) [in Chinese]. Zhonghua Er Ke Za Zhi [Chinese J Pediatr].

[18] Jia A, Wang Y, Sun W, Xiao B, Mu L, Wei Y (2017). Comparison of the roles of house dust mite allergens, ovalbumin
and lipopolysaccharides in the sensitization of mice to establish a model of
severe neutrophilic asthma. Exp Ther Med.

[19] Hamamoto Y, Ano S, Allard B, O'Sullivan M, McGovern TK, Martin JG (2017). Montelukast reduces inhaled chlorine triggered airway
hyperresponsiveness and airway inflammation in the mouse. Br J Pharmacol.

[20] Kumar M, Ahmad T, Sharma A, Mabalirajan U, Kulshreshtha A, Agrawal A (2011). Let-7 microRNA-mediated regulation of IL-13 and allergic airway
inflammation. J Allergy Clin Immunol.

[21] Sharma A, Kumar M, Ahmad T, Mabalirajan U, Aich J, Agrawal A (2012). Antagonism of mmu-mir-106a attenuates asthma features in allergic
murine model. J Appl Physiol (1985).

[22] Imamura K, Akimitsu N (2014). Long non-coding RNAs involved in immune responses. Front Immunol.

[23] Zhou L, Liu Y, Chen X, Wang S, Liu H, Zhang T (2018). Over-expression of nuclear factor-kappaB family genes and
inflammatory molecules is related to chronic obstructive pulmonary
disease. Int J Chron Obstruct Pulmon Dis.

[24] Wilson SJ, Rigden HM, Ward JA, Laviolette M, Jarjour NN, Djukanovic R (2013). The relationship between eosinophilia and airway remodelling in
mild asthma. Clin Exp Allergy.

[25] Hekking PP, Bel EH (2014). Developing and emerging clinical asthma
phenotypes. J Allergy Clin Immunol Pract.

[26] Shin IS, Jeon WY, Shin HK, Lee MY (2013). Effects of montelukast on subepithelial/peribronchial fibrosis in
a murine model of ovalbumin induced chronic asthma. Int Immunopharmacol.

[27] Tenero L, Piazza M, Sandri M, Azzali A, Chinellato I, Peroni D (2016). Effect of montelukast on markers of airway remodeling in children
with asthma. Allergy Asthma Proc.

[28] Yuksel B, Aydemir C, Ustundag G, Eldes N, Kutsal E, Can M (2009). The effect of treatment with montelukast on levels of serum
interleukin-10, eosinophil cationic protein, blood eosinophil counts, and
clinical parameters in children with asthma. Turk J Pediatr.

[29] Marques-Rocha JL, Samblas M, Milagro FI, Bressan J, Martinez JA, Marti A (2015). Noncoding RNAs, cytokines, and inflammation-related
diseases. FASEB J.

[30] Rignault-Bricard R, Machavoine F, Mecheri S, Hermine O, Schneider E, Dy M (2018). IL-3-producing basophils are required to exacerbate airway
hyperresponsiveness in a murine inflammatory model. Allergy.

[31] Han M, Hong JY, Jaipalli S, Rajput C, Lei J, Hinde JL (2017). IFN-gamma blocks development of an asthma phenotype in
rhinovirus-infected baby mice by inhibiting type 2 innate lymphoid
cells. Am J Respir Cell Mol Biol.

[32] Camargo CA, Gurner DM, Smithline HA, Chapela R, Fabbri LM, Green SA (2010). A randomized placebo-controlled study of intravenous montelukast
for the treatment of acute asthma. J Allergy Clin Immunol.

[33] Wang X, Liu B, Lu B, Zhang Y, Wang L, Li H (2017). Micro-invasive embedding combined with montelukast sodium for
children cough variant asthma: a randomized controlled trial [in
Chinese]. Zhongguo Zhen Jiu.

[34] Debelleix S, Siao-Him Fa V, Begueret H, Berger P, Kamaev A, Ousova O (2018). Montelukast reverses airway remodeling in actively sensitized
young mice. Pediatr Pulmonol.

[35] Maeba S, Ichiyama T, Ueno Y, Makata H, Matsubara T, Furukawa S (2005). Effect of montelukast on nuclear factor kappaB activation and
proinflammatory molecules. Ann Allergy Asthma Immunol.

[36] Song G, Zhang Y, Yu S, Lv W, Guan Z, Sun M (2019). Chrysophanol attenuates airway inflammation and remodeling
through nuclear factor-kappa B signaling pathway in asthma. Phytother Res.

[37] Lai J, Mei ZL, Wang H, Hu M, Long Y, Miao MX (2014). Montelukast rescues primary neurons against Abeta1-42-induced
toxicity through inhibiting CysLT1R-mediated NF-kappaB
signaling. Neurochem Int.

[38] Ezegbunam W, Foronjy R (2018). Posttranscriptional control of airway
inflammation. Wiley Interdiscip Rev RNA.

[39] Zhang HJ, Wei QF, Wang SJ, Zhang HJ, Zhang XY, Geng Q (2017). LncRNA HOTAIR alleviates rheumatoid arthritis by targeting
miR-138 and inactivating NF-kappaB pathway. Int Immunopharmacol.

